# Lexical Context Effects During Reading: The Impact of Vocabulary Knowledge and Word Frequency

**DOI:** 10.3390/jemr19050095

**Published:** 2026-08-27

**Authors:** Ashley N. Abraham, Jocelyn R. Folk

**Affiliations:** 1 Psychology Department, School of Education, Health, and Human Behavior, Southern Illinois University—Edwardsville, 1 Hairpin Drive, Edwardsville, IL 62026, USA; 2 Department of Psychological Sciences, College of Sciences and Humanities, Kent State University, 1075 Risman Drive, Kent, OH 44243, USA; jfolk@kent.edu

**Keywords:** visual word recognition, eye movements, word frequency, lexical quality, vocabulary, context effects, reading, literacy, reading comprehension

## Abstract

Skilled readers are thought to rely on bottom-up processing to activate word meanings in long-term memory from their form (i.e., spelling or sound) in a process described as ‘context-free’ lexical processing. However, other research suggests skilled readers recognize words faster when they appear in predictable and/or plausible contexts, despite efficient bottom-up processing. Research on individual differences in skilled comprehension suggests skilled readers only use context to support word recognition when bottom-up processing is slow. Therefore, highly skilled readers may not rely on context, but less-skilled readers may continue to rely on context during word recognition due to poor bottom-up processing. The current study investigated whether individual differences in lexical quality would influence contextual processing during word recognition. Participants’ eye movements were tracked as they read sentences containing either a high-frequency (e.g., baby) or low-frequency (e.g., elbow) target word preceded by either a related prime word (mother–baby) or an unrelated control word (teeth–elbow). Results show relatedness had a significant effect on readers across the range of lexical knowledge; however, the specific effect depended on both vocabulary and target word frequency. The results suggest that context supports word recognition and comprehension when readers have adequate vocabulary.

## 1. Introduction

For skilled adult readers, words seem to be effortlessly, even obligatorily lifted from the page, transformed into something meaningful, and woven seamlessly into the unfolding text. It is only when this automatic lexical process of accessing word meanings from their printed forms breaks down that the skilled reader may become consciously aware of any difficulty recognizing words. As such, much attention has been devoted to understanding how readers develop automatic word recognition, learning to retrieve word meanings automatically from long-term memory and becoming skilled readers that unconsciously engage efficient lexical processing of word form, word meaning, and integration into the text [1,2,3,4,5]. Research on skilled adult readers has demonstrated that differences in underlying lexical processing can lead to differences in how a word is recognized and manifest as differences in reading comprehension [6,7,8,9]. According to the Reading Systems Framework (RSF), these differences can be attributed to differences in the *quality* of orthographic (spelling), phonological (sound), and semantic (meaning) representations in LTM [7]. Of particular interest is whether skilled adults use contextual information to support word recognition when it is slowed by inefficient lexical processing.

### 1.1. Context Effects in Skilled Adult Reading

A consistent finding in the literature is that when words appear in related context, they are processed faster than when they do not appear in related context [3,10,11,12,13,14,15,16,17]. This is problematic given the common characterization of skilled reading as relying on ‘context-free’ word recognition. However, when bottom-up lexical recognition is slow, the top-down impacts of context may be observed [3]. For skilled adults, this can mean, for example, when encountering an unknown or unfamiliar word, prior context may be available to influence early stages of the reading process. Despite the consistency of this finding, there is little agreement about how context facilitates reading and whether it operates at a word level and/or a later integrative level [18,19]. One reason for this disagreement is that there are two different types of sentence context discussed in the literature, lexical and message context, whose putative mechanisms occur at different levels of word processing.

Message context refers broadly to the relational elements, usually within a sentence, often engaging inferential processing and other cognitive domains (e.g., working memory, syntactic parsing, and general world knowledge). Lexical context, however, refers specifically to word-to-word relationships, relying on strong semantic associations encoded within the lexicon (e.g., doctor primes nurse) [20,21,22,23,24]. Whether message-level context can influence lexical word recognition remains an open question due in part to the difficulty in disentangling it from lexical context. However, lexical context effects are thought to arise from spreading activation entirely within the lexicon [20]. If a reader encounters the word ‘doctor’ early in a sentence, they may be faster to read the word ‘nurse’ later because of pre-activation of their shared semantic network. Previous findings regarding this intralexical priming mechanism as a mechanism of contextual facilitation in skilled reading have been mixed [15].

One of the reasons why the effect of lexical context on word recognition is unclear is because much of the evidence in support of this mechanism of contextual facilitation comes from studies that do not employ naturalistic reading tasks, such as naming and lexical decision [20,21,22,23,24]. Additional evidence suggests that intralexical priming from lexical context may occur only when the target word is supported by coherent message-level context [14]. Traxler et al. [15] investigated this relationship between lexical-level and message-level context effects in a series of experiments using a natural reading task but found minimal support for intralexical priming through spreading activation. In their experiments, prime type (associative, identity, and synonym) and plausibility were manipulated. They found that identity (i.e., same word repetition) primes, but not associative or synonym primes, produced facilitation in measures of early processing, indicative of an intralexical priming effect. However, their plausibility manipulation allowed for an examination of message-level context effects. They found that target words (e.g., axe) were read faster in plausible message-level contexts (e.g., *The lumberjack carried the axe*…) than implausible message-level contexts (e.g., *The lumberjack chopped the axe*…), regardless of prime type. Despite the presence of lexical associates in both sentences (lumberjack–axe), and the instantiation of a consistent schema (lumberjacks are often found with axes), facilitation for the target word (axe) occurred only when the message-level context was plausible (lumberjacks chop wood, not axes, but they do carry axes). The researchers suggest that their results are best reconciled with a situation model of priming wherein facilitation occurs for targets that are easily integrated with the text representation (i.e., plausible targets), but they leave open the possibility that intralexical priming may be a contributor to contextual facilitation during natural reading even if its impact is minimal [11,16,17,25]. These findings suggest that message-level plausibility, not intralexical priming, may be a mechanism by which context facilitates processing. These conclusions are based, however, on results averaged across readers of varying skill. Recent research addressing individual differences in reading comprehension suggests that context may differentially impact readers who vary in underlying reading component skills.

### 1.2. Individual Differences in Context Effects

Recent research regarding individual differences has centered on the lexical quality hypothesis (LQH). The LQH posits that a single ‘word’ is comprised of orthographic, phonological, and semantic representations, which are stored in the lexicon. Each of these constituent representations can be more or less accurate and specific. Each constituent can also be more or less connected to the others. Thus, a high-quality representation for a single word consists of accurate, specific, and well-connected constituent representations that are automatically activated by the activation of the others. Low-quality representations may be due to inaccurate, underspecified, and/or disconnected word knowledge. According to the RSF, differences in the quality of word knowledge are associated with qualitative differences in how word representations are activated in LTM [7]. Research on context effects with the LQH has led to a general conclusion that less skilled readers, those who do not have high-quality representations for a large number of words, rely more on context to support word recognition than experts or more skilled readers. This raises questions about what it means to ‘rely on’ context with respect to lexical word recognition, what type of context readers may be relying on, and which domains of word knowledge or reader-specific knowledge may influence activation of meaning from text.

Highly skilled readers quickly recognize words through efficient bottom-up processing and do not need the support of top-down context [3]. In contrast, the “sluggish,” inefficient bottom-up processing associated with less-skilled readers provides time for context to influence word recognition [3,9,26,27,28]. Research shows that highly skilled readers do not benefit from having context available when reading individual words; instead, word recognition occurs rapidly both with and without context [9,26]. This has led to the assertion that highly skilled readers rely less on context for word recognition than less-skilled readers.

Andrews and Bond [26] investigated skilled readers’ reliance on context using a memory probe task. Highly skilled and less-skilled college-aged adult readers read ambiguous words in informative sentence contexts (e.g., She danced all night at the ball) and then responded to a memory probe word that was not presented in the sentence but was related to one of the ambiguous word’s meanings (*WALTZ/THROW*). Highly skilled readers, particularly those with high spelling skill, were less likely to falsely report that a contextually related probe (e.g., *WALTZ*) had been present. Less-skilled readers were more likely to falsely report this, suggesting they used sentence context to support meaning activation for contextually related probe words, leading to a “false memory.” The authors suggest that highly skilled readers recognize words using bottom-up lexical processing independent of context, while less-skilled readers continue to rely on context to compensate for inefficient lexical processing well into adulthood. This research is part of a growing body of literature suggesting that top-down contextual processing does not influence word recognition for highly skilled readers, but that less-skilled adult readers rely on contextual information to support inefficient bottom-up, lexical word recognition [3,26,27,28,29].

These findings are consistent with predictions derived from the lexical quality hypothesis (LQH). According to the LQH, highly skilled readers have developed strong and precise word knowledge representations that support context-independent word recognition from lexical processing [28]. The word knowledge representations of highly skilled readers are thought to be very well connected, such that activation in one knowledge component produces automatic activation of other knowledge components. When a highly skilled reader encounters a printed word form, the orthographic representation is quickly activated, which then automatically activates the semantic (and phonological) representation through bottom-up processing. Fast and synchronous activation among constituent components leads to efficient word recognition, which in turn allows for fast and efficient higher-order processing, such as integration and text comprehension. High-quality word representations are formed over time through reading experience.; Highly skilled readers are characterized as having high-quality representations for a greater number of words than less-skilled readers, allowing them to recognize individual words quickly and accurately with little influence from context.

### 1.3. The Reading Systems Framework

The reading systems framework (RSF) is a broad account of the reading system that incorporates the LQH and positions the lexicon as the central ‘pressure point’ situated between the word identification system and the comprehension system [7]. Within this system, individual differences are attributed to differences in the quality of representations stored in the lexicon. When a reader encounters a word, sub-lexical and lexical processes in the word identification system are engaged, followed by selecting a lexical item from among the candidates currently active in the lexicon. Critically, prior context incorporated into the active situation model can also influence lexical access. The RSF describes a backward memory mechanism by which readers integrate new words into developing text representations: as lexical entries become active candidates for word recognition, their representations activate associated areas of long-term memory. When these activated areas “resonate” with areas activated by the text representation, word recognition may be facilitated [30].

As an alternative to traditional, modular accounts of visual word recognition processes (i.e., interactive-activation, dual-route cascaded models), the RSF relies on an interactive, distributed, connectionist cognitive architecture [3,31]. In this view, word recognition is not separate from contextual processing. Instead, word recognition is achieved when activation within the lexicon settles to a stable state characterized by congruent activation of high-quality constituents and limited interference from other sources. If lexical activation does not stabilize quickly due to insufficient word knowledge (i.e., based on bottom-up processing of lexical information), feedback from top-down situational model priming can also activate relevant knowledge within the lexicon.

The RSF is similar to more traditional accounts of word recognition, such as the interactive-compensatory model, in that when bottom-up access to meaning is slow, top-down processes can provide additional support [3]. However, because the RSF uses a connectionist architecture, the distinction between bottom-up lexical processing and top-down contextual processing is blurred. Instead, partially overlapping word identification and word-to-text and/or text-to-word integration processes work in conjunction. In this view, if automatic word recognition from the visual input is slow, contextual information in the currently active situation model can feedback activation to associated areas of the lexicon. Assuming a level of text coherence, when information from context stored in the situation model compliments the active contents of the lexicon, both identification and integration may be facilitated. As such, the RSF allows for specific predictions regarding reader-specific lexical knowledge and text-specific context. In addition, the RSF make specific predictions about the interaction between lexical quality and other word-specific factors.

One of the most reliable of these word-specific factors is frequency, which influences the earliest stages of lexical processing [9,32]. High-frequency words, those that occur often in print, are read more quickly than low-frequency words [32]. The RSF predicts that this typical frequency effect will be reduced within the highly skilled reader population because these readers will have developed high-quality representations for more words, including those that are low frequency. Ashby et al. [9] tested this hypothesis specifically and found that highly skilled readers, assessed for reading comprehension, did indeed show reduced frequency effects and were less susceptible to predictability, while less-skilled readers showed typical frequency effects and a greater influence of predictable context. However, Ashby et al. only assessed reading comprehension, a skill that combines several cognitive processes (e.g., working memory and inferencing) and reading strategies. Recent research suggests that more precise measures of lexical quality, such as spelling and vocabulary knowledge, more directly tap the quality of lexical representations [6,8,33,34,35,36].

Andrews and Reynolds [36] examined the influence of lexical quality on contextual processing. According to the RSF, the resonance mechanism predicts facilitation for both co-occurring (i.e., intralexical priming) and predictable words. Co-occurrence captures word-to-word relationships much like semantic relatedness but is determined by the frequency with which two words appear in close proximity within a large text corpus [37], rather than semantic relatedness. To test these predictions, researchers assessed spelling skill, an indicator of orthographic precision, and vocabulary skill, an indicator of semantic knowledge and semantic flexibility, and investigated lexical co-occurrence probability and sentence predictability during a natural reading task. Their results suggested that co-occurrence and predictability are independent contextual processes: co-occurrence facilitated word recognition for all readers, evident in faster initial reading times in sentences with high versus low co-occurrence rates, but less-skilled readers were unable to make use of these co-occurring relationships to support integration, instead being more influenced by predictability. Integration for highly skilled readers was facilitated by co-occurrence regardless of predictability. However, this study used only low-frequency verb–noun pairs, and the highly related pairs were themselves predictable regardless of sentence context predictability (e.g., wreaked havoc vs. unleashed havoc). This could explain why co-occurrence did not interact with skill as predicted from the LQH, later subsumed under the RSF: even less-skilled readers are likely to be familiar with the idea of “wreaking havoc,” and it is difficult to imagine what, other than havoc, one might wreak. The facilitation observed from lexical co-occurrence in this study could, therefore, potentially reflect predictability rather than lexical processing. Together, previous research predicts that lexical–semantic relatedness should facilitate, or at worst leave unaffected, the recognition and integration of a target word, with the degree of facilitation depending on reader-specific lexical quality and word frequency.

More recent research investigating the effects of reader skill examined many reading measures associated with lexical quality to further explore the finding of a reduced frequency effect among highly skilled readers. Lee et al. [38] assessed a battery of reading-related skills, including vocabulary, spelling, print exposure, and reading comprehension, in skilled adult readers who then read either a high- or a low-frequency target noun in single, unpredictable sentence frames. They found that when measures were included in models as unique factors, higher vocabulary and reading comprehension scores generally predicted reduced frequency effects in gaze duration, consistent with the LQH. A principal component analysis found vocabulary skill, specifically as measured by the Nelson–Denny Reading Test (NDRT), was associated with a general “lexical proficiency” factor, including spelling and other reading ability measures. Interestingly, the comprehension subtest of the NDRT was not found to moderate frequency effects independent of the vocabulary subtest for any of their word-based measures and was not significant in interaction models that included other predictors. The authors suggest that the reduced frequency effects observed in early reading measures associated with the NDRT composite score were driven by vocabulary, not comprehension, given the loading of NDRT vocabulary on to the lexical proficiency factor independent of comprehension. Together, their results suggest that early differences in word recognition associated with lexical quality may be present but harder to reliably detect and more sensitive to task demands and measures. Moreover, their results indicate that vocabulary skill is a critical determinant of lexical processing and frequency effects.

### 1.4. The Current Study

To investigate the effect of lexical context on word recognition, and to test the lexical quality predictions of the RSF, the current study examines the interaction of lexical co-occurrence with word frequency and how these factors may be differentially impacted by reader skill. Conflicting findings regarding intralexical priming from semantic associates during reading, as well as research suggesting that lexical quality differences influence how words are recognized in context, necessitates further investigation of intralexical priming as a potential mechanism of contextual facilitation. Moreover, it is important for future research that the contributions of lexical context during word recognition are established in order to disentangle this effect from message-level context effects.

The present study examines whether skilled adults continue to rely on, or perhaps default to, a more contextually pervious word recognition process when lexical processing is “sluggish.” This sluggishness may result from the reader having poor-quality word knowledge, properties of the word, or the characteristics of the context in which it appears. Therefore, to examine the differential effects of lexical quality during word recognition, the current study measured reader-specific spelling and vocabulary skills and manipulated semantic co-occurrence within target word frequency conditions. Eye movements were tracked during a natural reading task to approximate a typical reading situation, and effects were examined separately in early (e.g., first fixation duration and gaze duration) and late (e.g., total reading time and regressions) eye movement measures to localize any effects of relatedness to lexical or post-lexical processing stages. Based on the theoretical accounts reviewed above, the present study tested the following hypotheses:

**H1.**  *Consistent with resonance-based accounts incorporated into the RSF, lexical–semantic relatedness was predicted to facilitate recognition and integration of target words across all readers and frequency conditions.*

**H2.**  *Consistent with the LQH, the influence of relatedness was predicted to depend on reader vocabulary skill and target word frequency, such that readers with lower-quality lexical representations (lower vocabulary skill) would show a different pattern of relatedness effects for related words than readers with higher-quality representations.*

**H3.**  *If relatedness effects on word recognition are lexically based rather than arising from post-lexical integration failure, these effects should emerge as faster reading times on initial eye movement measures (e.g., first fixation and gaze duration) rather than emerging only in late measures (e.g., total reading time and regressions).*

## 2. Materials and Methods

### 2.1. Participants

One hundred and fifty-eight students from Kent State University participated for course credit. Based on the best practices recommended by [39], 150 participants was determined to yield sufficient power for detecting a small to medium effect size for fixed effects, with 2 levels and 40 observations at each level. All participants were required to have normal or corrected vision, speak English as their first language, and have no known reading disabilities. Participants were drawn from a college-aged sample. No other demographic information was collected from participants. Two additional unique samples of 50 students participated in norming studies.

### 2.2. Stimuli

Forty high-frequency and forty low-frequency target words were selected for experimental sentences. CELEX word frequency counts were used to determine high- and low-frequency target words [40]. Words were considered low frequency when they occurred less than 30 times per 1 million words, and high-frequency words were those that occurred more than 70 times per 1 million words. All target words were nouns ranging from 5 to 7 letters. All 80 target words were embedded in sentences. Each sentence also contained a prime word before the target word. Latent semantic analysis (LSA), a measure of lexical co-occurrence, was used to determine the strength of the semantic relationship between prime and target words [41]. LSA is an analysis of the degree of semantic relatedness between individual words, phrases, and texts taken from 444 works of literature and includes more than 57 million words. In this technique, each word is represented as a vector, and relatedness is determined by the distance between vectors within a 300-factor multidimensional semantic space. Semantic relatedness ranges from −1.0 to +1.0. A score of +1.0 indicates perfect semantic relatedness (identical words). Scores above +0.30 indicate strong semantic relatedness. An a priori cut-off of +0.50 was used to select highly related prime–target pairs [42].

All prime words were high-frequency nouns ranging from 4 to 7 letters. Prime words and target words were separated by less than 10-character spaces. Experimental sentences were constructed so that only the prime word was related to the target (see Table 1). Additional LSAs were conducted to assess the semantic relationship between the target word and individual words prior to the target and to assess relatedness between the target and the entire preceding phrase. This was to ensure that no word or combination of words, other than the related prime, was related to the target word. In the unrelated condition, related prime words were replaced with unrelated control words that did not have a strong semantic relationship with the target word, as indicated by an LSA score of less than or equal to +0.20. Thus, control sentences were constructed so that only prime–target relatedness was manipulated.

Each participant viewed only one version, related or unrelated, of each target word. Two counterbalanced stimulus lists were constructed such that each target word appeared in the related condition on one list and in the unrelated condition on the other. Within each list, participants read 40 high-frequency and 40 low-frequency words, embedded in 20 related and 20 unrelated sentences at each frequency level such that participants saw 40 related and 40 unrelated trials overall. Participants were alternatingly assigned to a counterbalancing condition, ensuring that each target word appeared approximately equally often in the related and unrelated conditions across the full sample.

#### Material Norming Studies

A unique group of 50 participants completed a CLOZE task to ensure that the target words were not predictable from the prior sentence context. To complete the CLOZE task, participants viewed the experimental and control sentences up to but not including the target word. Participants were asked to provide the next word in the sentence. An a priori cut-off of 20% was used to determine target word predictability. Any target word predicted by more than 20% of participants was replaced with a nonpredictable target in experimental sentences. Overall, the probability of predicting a target word was low (*M* = 0.03, range = 0.00–0.18). Target predictability was similar across high-frequency (*M* = 0.03, range = 0.00–0.14) and low-frequency targets (*M* = 0.02, range = 0.00–0.18) and across related (*M* = 0.03, range = 0.00–0.18) and unrelated targets (*M* = 0.02, range = 0.00–0.14).

A separate group of 50 participants completed a norming study to ensure that target words in both conditions fit equally well within the overall sentence context. In this task, participants viewed the entire sentence and rated the degree to which the target word fit with the surrounding context. A 5-point Likert scale was used to assess target word sentential fit. Sentential fit was matched across conditions so that target words were equally appropriate in both the related prime (*M* = 3.86, range = 2.24–4.72) and unrelated prime sentences (*M* = 3.66, range = 2.08–4.44). In addition to experimental stimuli, 40 filler sentences were included in the reading task. Filler sentences were immediately followed by comprehension questions to ensure that participants were reading for comprehension.

### 2.3. Apparatus

Data were collected using an SR Research Eyelink 1000 Plus eye tracker (SR Research Ltd., Ottawa, ON, Canada) with a sampling rate of 1000 Hz [43]. Sentences were presented on a 21.5-inch iMac Retinal Display video screen. Participants were seated approximately 60 cm away from the screen. Reading was binocular. However, eye movements were recorded from the right eye only. One degree of visual angle was equal to 2.4 letters.

### 2.4. Procedure

After participants provided consent, they began a skills assessment. The skills assessment included measures of vocabulary and spelling skill knowledge. Participants had 15 min to complete the vocabulary subtest of the Nelson–Denny Reading Test [44]. Participants were notified of the time remaining at 10 min remaining, 5 min remaining, and 1 min remaining. Then, participants completed a spelling assessment. The spelling recall assessment is comprised of 20 items that capture the variability in skilled spelling [45] and normed using two independent samples of participants from two universities [46]. To complete the assessment, participants listened to an audio recording of a word and were asked to provide the correct spelling to the best of their ability on an answer sheet. Participants also completed a spelling recognition measure. The recognition measure consisted of 50 commonly misspelled words. Twenty-five of the items were presented with the correct spelling and the other twenty-five were misspelled. Participants were asked to circle the misspelled words. The spelling recognition measure was self-paced. Both spelling measures were used to create a standardized composite score of overall spelling ability [26].

After completing the skills assessment, participants began the reading task. Participants were informed about the eye tracking procedures. They were told to rest their chin on a support and place their forehead on a stabilizing bar to reduce head movements. The experimenter then calibrated the eye tracker. During calibration, participants were told to follow a white circle with their eyes as it moved to nine different points on the display screen. This ensured an accurate gaze location. Calibration was considered successful when the degree of visual angle was less than 0.50 degrees for all nine points. The experimenter ensured that calibration was accurate prior to every trial. If the degree of visual angle error exceeded 0.50, the experimenter recalibrated before continuing to the next trial.

Participants read a total of 120 sentences: 80 experimental sentences and 40 filler sentences with comprehension questions. The order of sentence presentation within each list was completely randomized. Each trial began with participants looking at a fixation point indicated by a white circle on the screen. The fixation point is where the first word of the sentence appeared. When participants looked at this point, the experimenter presented the next sentence. Participants were instructed to read silently for comprehension at their own pace. They were told to press a button when they finished reading the sentence. To ensure that participants were reading for comprehension, filler sentences were included and followed by a “yes” or “no” comprehension question. Participants were told to press one button to respond “yes” and another button to respond “no.” Participants who failed to answer comprehension questions with 80% accuracy or above were removed from the dataset prior to analysis. Before beginning the reading task, participants viewed six practice sentences and six comprehension questions to familiarize them with the procedure prior to reading the experimental items. Participants were debriefed after completing the reading task. The experiment took approximately 45 min to complete.

### 2.5. Individual Difference Measures

The vocabulary subset of the NDRT was used as a proxy measure of the lexical quality of the semantic component of word knowledge. Vocabulary skill is associated with both higher-order comprehension processes and bottom-up lexical processing [29,34,47]. Spelling skill is an indicator of the orthographic component of word knowledge and, therefore, critically associated with bottom-up lexical processing [26,33,47,48]. Raw means and standard deviations for the individual difference measures are reported in Table 2. Vocabulary scores were standardized prior to analysis. A spelling composite (Scomp) score was computed from standardized scores of spelling recall and spelling recognition [48,49]. First, the spelling recall proportion correct was converted to z-scores (zRecall). Second, d-prime, a standardized score of response sensitivity, was calculated. This was done to account for true positive responses (‘hit’) and false positive responses (‘false alarms’). The proportion of true positive responses (HIT) and the proportion of false positives (FA) were converted to z-scores, zHIT and zFA, respectively. D-prime was calculated by subtracting zFA from zHIT:

d-prime = zHIT − zFA.

Finally, d-prime and zRecall were averaged to create the composite spelling score, Scomp.

### 2.6. Data Cleaning Procedures

Average accuracy on comprehension questions was 92%. Four participants were removed from analyses for scoring less than 80% on comprehension questions. Individual fixations less than 100 ms and fixations more than 1000 ms were excluded from analysis because individual fixations outside of this range are not representative of typical eye movement behavior during reading [32]. Trials during which track loss occurred were also excluded from analysis. This resulted in 3.3% of data lost due to track loss and fixations exceeding the typical range. Trials during which the related or unrelated prime word (n) was skipped were excluded from analysis. To minimize data loss and to account for parafoveal preprocessing of the prime (Readers are sometimes able to recognize words on the preceding fixation during the parafoveal preview period. This occurs when readers recognize the fixated word and shift their attention, but not their eyes, to the upcoming word. If this word can be recognized quickly, making a fixation is not necessary and readers may skip the word [50]. In the context of the current study, including trials with a fixation on *n* or *n* − 1 allows for parafoveal processing of the prime and word recognition in the absence of a fixation on the word.), participants who made a fixation on the preceding word (*n* − 1) were not excluded from analyses. Thus, only trials during which the participant skipped both the prime (*n*) and the word preceding it (*n* − 1) were omitted, resulting in the exclusion of 223 trials (4% of trials). The critical regions of interest for each included the target word and post-target sentence region up to but not including the sentence-final word. Two measures of initial processing were included: first fixation duration (FF), the duration of the first fixation in an AOI (area of interest), and gaze duration (GD), the sum of all first-pass fixations in an AOI, including first-pass refixations. Three measures of late processing were included: total time (TT), the sum of all fixations in an AOI, a measure that includes all initial reading and rereading, regressions in (RI), defined as the probability of returning to a prior AOI from a later one, and regressions out (RO), defined as the probability of leaving the currently fixated AOI to return to a prior one.

Analyses were conducted within the R Environment for Statistical Computing v.3.6.3 [51] using the lmer function of the lme4 package to model fixed effects and random effects of both subjects and items. Continuous measures of eye movement behavior (first fixation, gaze duration, and total time) were log transformed and analyzed with linear mixed effects models (LMMs). Measures of fixation probability (regressions) were analyzed with generalized linear mixed effects models (GLMMs) using the glmer function of lme4. For each dependent measure, two models were fit. The overall model included frequency and prime conditions, and their interaction, as fixed effects, testing whether relatedness impacts processing, as predicted by a spreading activation account (H1). The skill model added vocabulary and spelling skill as continuous predictors along with all interactions, testing whether the effect of relatedness on processing depends on lexical quality, as predicted by the RSF (H2). For H3, that relatedness is a lexical effect, we examined the interaction of word with relatedness in early eye movement measures (FF and GD) in the overall and skill models.

All models were structured to allow for the maximal random effects structure permitting convergence [52]. All continuous predictors were centered and scaled prior to analysis. Final models included random intercepts for subjects and items, with random slopes included where they improved fit without convergence. The overall model for FF and the skill model for GD retained a random slope for frequency by subject, while all remaining models converged with random intercepts for subjects and items only. Where the skill model revealed a significant three-way interaction among frequency, prime condition, and vocabulary skill, follow-up analyses were included. For follow-up analyses, the vocabulary measure was converted to a factor with three levels of skill determined by standard deviation from the mean. Participants who scored 1 standard deviation below the mean on the vocabulary measure were classified as below average, participants 1 standard deviation above the mean were considered above average, and participants scoring between +1 and −1 standard deviations from the mean were considered average.

## 3. Results

### 3.1. Individual Differences

Descriptive statistics for the individual difference measures are reported in Table 2. The spelling composite (Scomp) and vocabulary scores were positively correlated: *r* = 0.34, *p* < 0.001, and spelling recall and spelling recognition scores were also significantly correlated: *r* = 0.54, *p* < 0.001 (see Table 3).

### 3.2. Overall Effects of Frequency and Relatedness

In the overall models of initial processing, not including reader skill, significant effects of frequency on target word FF and GD were found, consistent with predictions (see Table 4). FF durations were significantly shorter for high-frequency target words (*M =* 237, *SE =* 2.0) than low-frequency words (*M =* 248, *SE =* 2.0; *β* = −0.02, *SE* = 0.01, *t* = −2.22, *p* < 0.05; marginal R^2^ = 0.004, conditional R^2^ = 0.13). Gaze durations were also significantly shorter for high-frequency (*M =* 257, *SE =* 2.9) target words than low-frequency targets (*M =* 278, *SE =* 2.9; *β* = −0.04, *SE* = 0.01, *t* = −3.04, *p* < 0.01; marginal R^2^ = 0.01, conditional R^2^ = 0.15).

The effect of frequency on target word TT, a measure which combines initial and late processing, was trending toward significance (*β* = −0.03, *SE* = 0.02, *t* = −1.77, *p* = 0.08; marginal R^2^ = 0.004, conditional R^2^ = 0.18) (see Table 5). This suggests that readers spent less time rereading high-frequency targets (*M* = 343, *SE* = 5.3) than low-frequency target words (*M* = 363, *SE* = 5.2). The probability of making a regression into the target word was not significant.

There was a trend toward a significant effect of target word frequency on post-target GD (*β* = −0.09, *SE* = 0.05, *t* = −1.77, *p* < 0.08) (see Table 6). The overall model showed a significant interaction of frequency and prime condition on log-transformed TT in the post-target region (*β* = 0.07, *SE* = 0.03, *t* = 2.08, *p* < 0.05), indicating that when targets were low frequency, readers spent less time in the post-target region following a related prime than an unrelated prime word. The probability of making a regression out of the post-target regions was not significant.

### 3.3. Effects of Lexical Quality, Frequency, and Relatedness

When spelling and vocabulary were added to models, they contributed additively, such that being a more skilled reader was generally related to being a faster reader for main effects of spelling and vocabulary skill (see Table 7, Table 8, Table 9, Table 10, Table 11 and Table 12 for full model results). Of critical interest are the interactions of skill with frequency and prime conditions, particularly in initial processing measures of FF and GD.

#### 3.3.1. Initial Processing of Target Words

**Figure 1:**
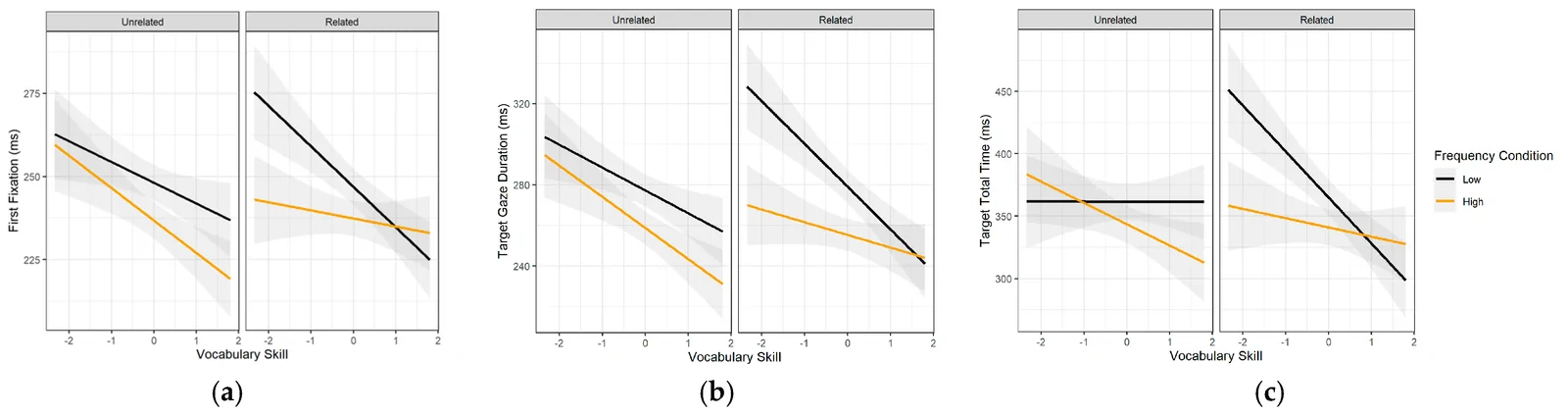
Target word reading measures. Each panel presents untransformed total time data instead of the log-transformed data used in analyses. Shaded regions around the lines represent 95% confidence intervals. (**a**) Vocabulary skill ×frequency × relatedness interaction on FF duration, (**b**) vocabulary skill × frequency × relatedness interaction on GD, and (**c**) vocabulary skill × frequency × relatedness interaction on TT.

There was a marginally significant interaction of frequency, prime condition, and vocabulary skill (*β* = 0.01, *SE* = 0.01, *t* = 1.89, *p* = 0.06, $R_{m}^{2}$ = 0.03, $R_{c}^{2}$ = 0.13) on FF, indicative of a lexical effect (see Table 7). Notably, this interaction was significant in the gaze duration data (*β* = 0.01, *SE* = 0.006, *t* = 2.06, *p* < 0.05, $R_{m}^{2}$ = 0.04, $R_{c}^{2}$ = 0.16; see Table 8). This suggests that the frequency effects observed in the overall model are modulated by lexical co-occurrence and vocabulary skill (see Figure 1).

#### 3.3.2. Late Processing of Target Words

There was a significant interaction of frequency, prime condition, and vocabulary skill on the probability of regressing into the target word (*β* = 0.11, *SE* = 0.06, *t* = 2.05, *p* < 0.05; $R_{m}^{2}$ = 0.01, $R_{c}^{2}$ = 0.17; see Table 9). This suggests that the probability of making a regression to a high-frequency target increased with reader vocabulary skill across both relatedness conditions. However, the probability of regressing to a low-frequency target increased with vocabulary skill in the unrelated condition and decreased with skill in the related condition.

There was a significant interaction of frequency, prime condition, and vocabulary skill on target word TT (*β* = 0.02, *SE* = 0.01, *t* = 2.65, *p* < 0.01; $R_{m}^{2}$ = 0.02, $R_{c}^{2}$ = 0.18; see Table 10). Similar to the early effects on target word processing, a frequency effect was observed in the unrelated condition for those readers with the highest vocabulary skill but observed in the related condition for readers with the lowest vocabulary skill (see Figure 1).

#### 3.3.3. Post-Target Region

There was a significant interaction of spelling skill, prime condition, and frequency on post-target GD (*β* = 0.02, *SE* = 0.01, *t* = 2.14, *p* < 0.05; $R_{m}^{2}$ = 0.03, $R_{c}^{2}$ = 0.36; see Table 11). Results suggest that gaze duration in the post-target region was shorter when it followed high-frequency words and decreased as spelling skill increased, but the decrease was greatest in the unrelated prime condition.

There was also a significant interaction of frequency, prime condition, and vocabulary skill in post-target RO probability (*β* = 0.08, *SE* = 0.04, *t* = 2.10, *p* < 0.05; $R_{m}^{2}$ = 0.02, $R_{c}^{2}$ = 0.29; see Table 12). RO probability increased in the unrelated prime condition as vocabulary increased. In the related condition, the probability of a regression out of this region decreased for low-frequency words as vocabulary skill increased.

### 3.4. Post Hoc Analyses

The significant interaction in target gaze duration and total time measures indicated that the effect of word frequency on word recognition was dependent on whether the word was preceded by a related or unrelated prime word and the reader’s vocabulary knowledge. To observe the nature of the change in the frequency effect across relatedness conditions, a post hoc analysis of target gaze duration was conducted. Of interest was whether changes in the frequency effect were driven by faster reading times on high-frequency words, longer reading times on low-frequency words, or both. Although vocabulary skill was treated as a continuous predictor in the primary models, the vocabulary measure was converted to a factor with three levels (below average, average, and above average), determined by 1 standard deviation around the mean, to facilitate visualization and interpretation of this interaction.

**Figure 2:**
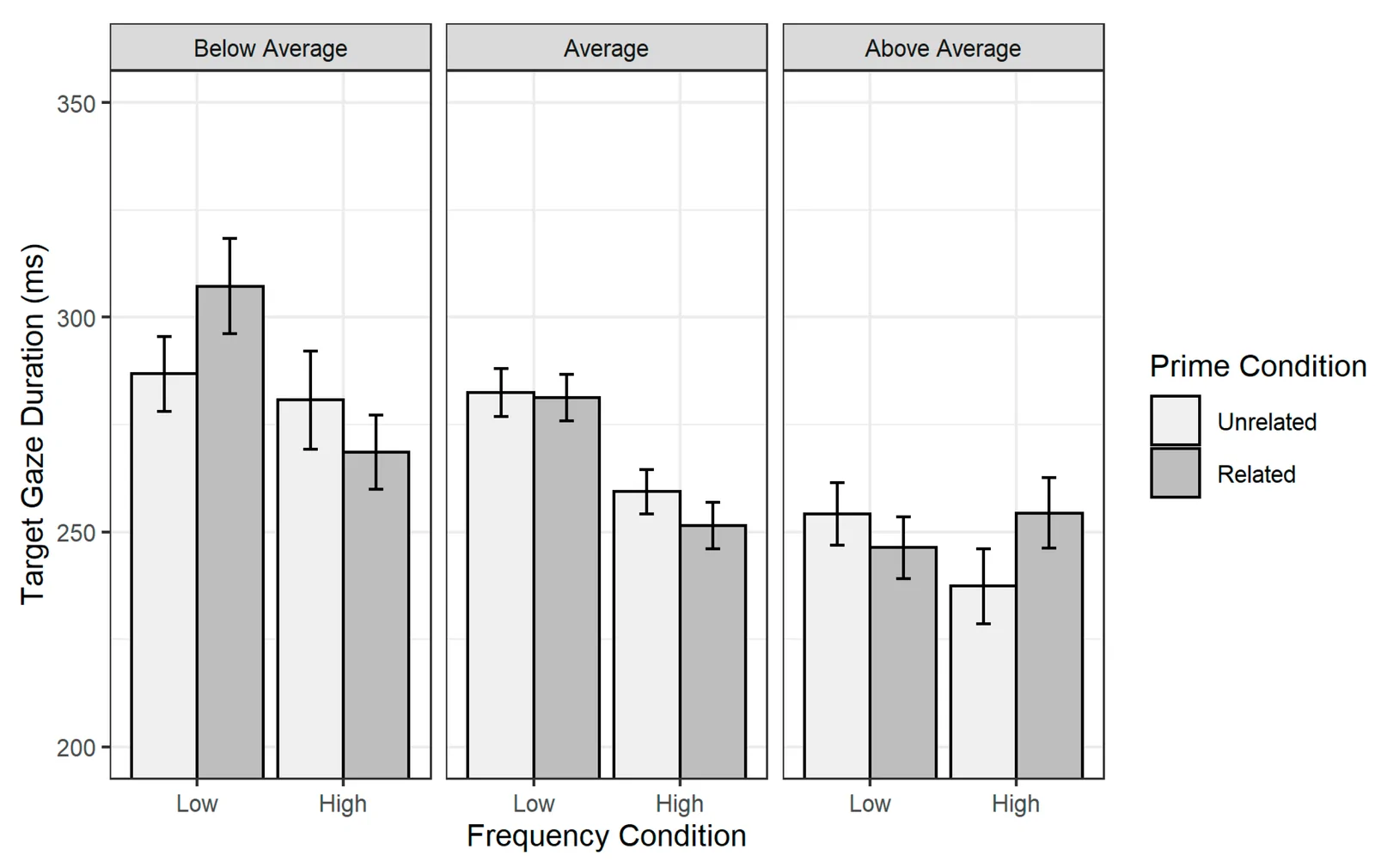
Gaze duration on the target word by condition and vocabulary group. The figure presents untransformed gaze duration data instead of the log-transformed data used in analyses. Groups represent 1 standard deviation around the mean. Error bars represent standard error.

Means and standard errors by vocabulary skill group for log-transformed target word gaze duration are presented in Table 13. The post hoc analysis indicates several significant differences between conditions within skill groups (see Figure 2).

For below-average readers, there was a significant difference in gaze duration between high-frequency related (HFRP) words and low-frequency related words (LFRP): *t*(361) = −2.93, *p* = 0.00, indicative of a frequency effect. There was also a significant difference between high-frequency unrelated words (HFUR) and LFRP: *t*(346) = −2.04, *p* = 0.04. No significant difference was found for LFRP and low-frequency unrelated (LFUR) target words or for HFUR and LFUR. The pattern of results suggests that the frequency effect observed in the related prime condition is due to an increase in reading time for LFRP relative to other conditions. Average readers demonstrated typical effects of frequency—gaze durations on HFUR words were significantly shorter than LFUR: *t*(1074) = −3.25, *p* = 0.00, and gaze duration in the HFRP condition was significantly shorter than in the LFRP condition: *t*(1077) = −4.22, *p* = 0.00. This suggests that relatedness does not influence word recognition for average readers over and above the effects of word frequency. For above-average readers, there was a marginally significant difference between HFUR and LFUR gaze durations: *t*(328) = 1.81, *p* = 0.07, suggesting a typical effect of frequency. However, there was also a marginally significant difference between HFRP and HFUR gaze durations: *t*(367) = −1.751, *p* = 0.08. Surprisingly, this finding suggests that the absence of the frequency effect in the related condition is not due to faster reading time in the LFRP condition but rather due to longer reading time in the HFRP condition.

The second post hoc analysis examined simple effects in log-transformed target word total reading time. Means and standard errors by condition and vocabulary skill group are presented in Table 14. The results for below-average and average readers are similar to those observed in the post hoc analysis of gaze duration. For below-average readers, there was a significant difference in total time for HFRP and LFRP: *t*(362) = −2.40, *p* = 0.02, and for HFUR and LFRP: *t*(347) = −2.10, *p* = 0.04. There was also a significant difference between LFRP and LFUR conditions: *t*(355) = 2.24, *p* = 0.03, indicating that below-average readers spent more time rereading related targets when they were low frequency. Average readers again showed typical frequency effects. Total time in the HFRP condition was significantly shorter than in the LFRP condition: *t*(1069) = −2.68, *p* = 0.01, and significantly shorter in the HFUR than the LFUR condition: *t*(1038) = −2.18, *p* = 0.03. For above-average readers, there was again a significant difference between the HFUR and LFUR conditions: *t*(365) = −1.84, *p* = 0.07, suggesting a typical effect of frequency. The difference between HFRP and HFUR was not significant. However, there was a significant difference between the LFRP and LFUR conditions: *t*(392) = −1.97, *p* = 0.05, indicating that above-average readers spent less time rereading in the LFRP condition, consistent with predictions.

## 4. Discussion

We investigated the extent to which a reader’s prior knowledge about words, specifically the quality of semantic knowledge, is associated with efficiently recognizing words in context during a natural, sentence reading task. The pattern of results suggests vocabulary knowledge is associated with *qualitative* differences in lexical processing, evident in early eye movement measures, and differences in comprehension processes, evident in late eye movement measures. By manipulating lexical–semantic relatedness and word frequency, we demonstrated differential effects for readers with high- and low-quality semantic knowledge, partially supporting predictions of the RSF [7]. High-quality knowledge was related to faster initial reading times and less rereading behavior overall. The interaction between lexical quality, co-occurrence, and frequency indicates that readers across the range of vocabulary knowledge are sensitive to lexical context, which sometimes facilitated recognition and comprehension, as predicted, particularly for those with high-quality semantic knowledge. However, contrary to predictions, low-quality semantic knowledge was associated with *longer* gaze durations (and a trend in FF) on low-frequency words in related context compared to the same low-frequency words in unrelated context. This suggests that for readers with the lowest-quality semantic knowledge, there was a higher processing demand associated with a large cost when recognizing low-frequency words in related context. This will be discussed in further detail below.

### 4.1. Individual Differences in Lexical Quality

The current study measured the vocabulary and spelling knowledge of a sample of skilled adult readers—college students. As such, participants were assumed to be a relatively skilled subset of the general population with experience reading a wide variety of texts independently for comprehension. Despite this characterization, a wide range of vocabulary and spelling skill was observed in the current sample. Although high spelling knowledge was associated with faster reading time overall and an increased probability of skipping the target word, the effect of spelling on all target word reading measures was additive. Thus, the differences in word recognition associated with spelling knowledge appear to be quantitative differences—highly skilled spellers are faster readers than less skilled spellers.

Although accurate orthographic representations, indexed by spelling skill, can enable the rapid activation of precise semantic representations, indexed by vocabulary skill, no significant interactions of spelling and vocabulary were observed, suggesting independent effects on word recognition. Instead, vocabulary knowledge uniquely interacted with frequency and relatedness on all target word reading measures, resulting in a pattern indicative of qualitative differences in word recognition processes. These results suggest a critical role for vocabulary knowledge when reading words in context.

### 4.2. Semantic Quality and Vocabulary Knowledge

The full fixed-effects structure of each critical model explained a modest proportion of variance (marginal R^2^ = 0.01–0.04 across models; Table 7, Table 8, Table 9, Table 10, Table 11 and Table 12), a model-level measure rather than an estimate specific to the three-way interaction. The interaction coefficients themselves were consistently small in magnitude (b = 0.01–0.02, with 95% CIs closely bounding zero; Table 7, Table 8, Table 9, Table 10, Table 11 and Table 12), indicating that these effects, while statistically reliable, are subtle. The interaction of vocabulary and frequency in gaze duration indicates that lexical relatedness information is available during the earliest stages of word recognition, even though the effect is small. A similar pattern was observed in first fixation durations, though this interaction was only marginally significant (*p* = 0.06) and should be treated as suggestive rather than establishing an early effect. Lee et al. [38] noted that early-measure interactions between word frequency and their lexical proficiency composite emerged consistently only when using intercept-only models. These subtle, early effects observed in our models were likely driven by two key methodological differences. First, whereas their study embedded targets in neutral sentence frames, the current study actively manipulated context to examine the reduction of the frequency effect *across* relatedness conditions. Second, prior research has relied on average-to-very-skilled samples. Our sample included a wider range of vocabulary skill, including a substantial group of below-average readers, which may have increased power to detect frequency-by-vocabulary interactions at earlier processing stages.

Of theoretical interest is the direction of this change in the frequency effect across context conditions and the full range of vocabulary skill. Based on the post hoc analyses of target gaze duration and total time, the change across conditions for below-average readers was due in large part to an *increase* in reading times for low-frequency, related words, not a decrease in reading times on high-frequency words, as predicted by lexical quality [28] or models of word recognition [4,20]. Additionally, an interesting but unpredicted marginal effect was observed for the above-average readers, suggesting the difference in the frequency effect is best characterized by longer reading times on high-frequency words, not faster reading of low-frequency, related words. Future research should examine whether this in indicative of rapid word-to-text integration processes predicted by the RSF [7].

### 4.3. Context Effects and the RSF

By accounting for reader-specific vocabulary knowledge, the current study demonstrated that readers sometimes took longer to read target words in lexically related context—this finding is inconsistent with traditional models of lexical context effects. Lexical context, defined by word-to-word associations, is thought to facilitate word recognition through spreading activation, wherein the currently active areas of long-term memory (LTM) yield activation in other, related areas of LTM. This automatic pre-activation of lexically related candidates facilitates word recognition through a speeded search process [20,53]. In this account, word recognition following a lexically related word should be faster than, or equivalent to, recognition of the same word when it is unrelated to previous words. However, in the current study, the interactive effects on target word reading measures are best characterized in terms of slower reading times, not faster. This demonstrates that lexically related context is not necessarily facilitative, and may instead make word recognition more demanding, a finding incompatible with an automatic spreading activation account of lexical context effects.

Alternative accounts of lexical context effects based on word-to-text integration processes make similar predictions regarding the facilitative effect of lexical context during word recognition. Previous research has suggested that situational model priming, wherein word recognition is facilitated when the word fits with the text representation, can account for lexical context effects [15]. Situation model priming was also incorporated into the reading systems framework (RSF), a broad view of the reading architecture that further accounts for the effects of lexical quality [7]. The RSF clarifies situation model priming by incorporating a backwards memory mechanism to describe how readers integrate new words into developing text representations. According to the RSF, as lexical entries become active candidates for word recognition, their representations activate associated parts of LTM. When these activated areas of LTM ‘resonate’ with the areas of LTM activated by the text representation, word recognition may be facilitated [30]. However, this model also predicts that word recognition and/or integration with the text representation should be easier when words are related. That is, in the experimental sentences from the current study, “He examined his *arms* and **elbow** when he fell yesterday,” and “He examined his *teeth* and **elbow** when he fell yesterday,” ‘elbow’ should be more easily incorporated into a situation model and/or should ‘resonate’ more strongly with the text representation when it follows the related prime word ‘arms’ than when it follows ‘teeth.’ A lexical quality account may propose that low-skill readers have difficulty forming a text representation and/or that low-quality semantic representations do not ‘resonate’ with text representations very strongly. However, in both instances, it should be no more difficult to recognize the word ‘elbow’ following ‘arms’ than ‘teeth’ nor should it be more difficult to integrate into the text representation. The finding that readers sometimes had longer reading times in the related condition suggests that recognizing and/or integrating words in lexically related contexts may actually be more challenging for some readers than recognizing and/or integrating the same words in unrelated contexts, inconsistent with predictions derived from situation model priming.

Integration accounts of context effects in reading attribute context effects to difficulty incorporating word meanings into a developing text representation after word recognition is complete. This differs from situation model priming in that integration occurs only after lexical processing has resulted in word recognition. Thus, integration accounts assert that longer initial reading times are due to a failure at a post-lexical stage rather than during lexical word recognition [54]. When integration failures occur, readers often regress to the place in the text where the difficulty occurred [55]. In the current study, there was a relatively low but significant probability of regressing from the post-target region into the target word (*M* = 13%, *SD* = 0.34). Thus, it does not appear as though most readers left the post-target region to return to the target word specifically. Instead, the probability of regressing out of the post-target region suggests that readers had more comprehension difficulty following low-frequency, related words, and this was most likely for readers low in vocabulary knowledge. Nonetheless, few readers experienced integration failure specific to the target word. Therefore, any integration failure is unlikely to be the result of lexical relatedness but may be due to post-lexical processing and attributed to more general comprehension difficulties.

Moreover, much of the evidence supporting early word-to-text integration effects during word recognition has been conducted with experimental manipulations of plausibility [56,57], predictability [58], message-level context [12,13,15,59], and discourse processing [60,61]. Manipulations of plausibility and predictability are necessarily confounded with message-level context and, therefore, not good candidates for isolating a lexical effect of context on word recognition. The current study maintained sentence plausibility and predictability such that within frequency conditions, sentences differed by a single prime word, but target words remained the same to minimize any effects from differences in message-level context and eliminate word-specific lexical factors. All prime words and targets words were nouns contained within the same sentence clause to ensure that effects were not attributable to syntactic processing (e.g., verb–noun linking) and/or to integrative processing that occurs across clause boundaries [7]. Therefore, the effects observed in early processing in the current study are not likely attributable to syntactic or contextual differences across conditions. Similarly, the demands on integrative processing are relatively consistent across conditions. Instead, because frequency has long been considered a lexical factor, the interaction of lexical context and vocabulary knowledge with word frequency during initial processing is indicative of a lexical effect.

### 4.4. Lexical–Semantic ‘Discord’ and Future Directions

Although current results suggest that the influence of lexical context depends on reader vocabulary skill and word frequency, the underlying cognitive mechanism is less clear. One alternative interpretation to spreading activation and post-lexical integration accounts discussed above is that the multiple sources of lexical input, which may produce relatively ‘harmonious’ lexical activation for some readers, may, however, produce for other readers a more discordant pattern and perhaps greater difficulty activating target word meanings. In the current study, target semantics can be activated by orthographic processing of the target, related words, or both. As both sources activate lexical semantics at different time points, different lexical candidates could compete for selection. An unintentional artifact of our experimental materials is that, for low-frequency targets in particular, the preceding context may activate a high(er)-frequency lexical candidate both directly and through that candidate’s own semantic association with the target. For example, arms may pre-activate hands, and elbow, becoming active via orthographic processing, may further produce activation for an unseen, related word, like hands, a high-frequency competitor, one which fits syntactically and thematically in the sentence. Thus, one possibility is that readers with less vocabulary knowledge to rely on may more readily activate a high-frequency competitor than a low-frequency target meaning under some conditions. A more direct test of this hypothesis could use the fast-priming paradigm developed by Rayner et al. [62]. This eye tracking technique presents a word within a sentence briefly (e.g., 25 ms) before changing to a different word. Though most readers are unaware of a change, it nevertheless impacts automatic lexical processing. Although our results suggest a critical role for semantic quality in skilled adult reading, we relied on a single measure, the NDRT vocabulary subtest, as our proxy for semantic lexical quality. Lee et al.’s [38] demonstration of the “jingle fallacy,” in which, in their study, two widely used comprehension tests were only moderately correlated, is a useful caution that measures sharing a construct label do not necessarily measure the same construct. It remains an open question whether the vocabulary-dependent pattern of relatedness effects observed here would replicate using a different vocabulary measure. The presence of a significant interaction in gaze duration, accompanied by a consistent but only marginally significant pattern in first fixation durations, suggests that lexical relatedness information may be available in the earliest stages of word recognition, and the specific effect of relatedness depends on the reader’s vocabulary knowledge. Any effect of lexical context is contingent on readers having previously encoded the word-to-word relationships captured by lexical co-occurrence. Readers across the dimension of vocabulary knowledge were sensitive to these lexically encoded relationships, and only those with high vocabulary knowledge benefited from this additional source of input. Lexically related context may, therefore, be more advantageous in supporting reading comprehension for those readers with a high level of vocabulary knowledge to rely on.

## Figures and Tables

**Table 1:** Example of experimental sentences in the related and unrelated prime conditions.

Condition	Prime–Target LSA	Sentence
Related	+0.78	He examined his *arms* and **elbow** when he fell yesterday.
Unrelated	+0.05	He examined his *teeth* and **elbow** when he fell yesterday.

**Table 2:** Descriptive statistics for individual difference measures.

Statistics	Vocabulary (Maximum = 80)	Spelling Recall (Maximum = 20)	Spelling Recognition (Maximum = 50)
*M*	56.05	3.9	39.67
*SD*	23.63	3.79	4.56
Range	7–76	0–17	28–49

**Table 3:** Correlations among individual difference measures ^1^.

	Vocabulary	zRecall	d-prime	Scomp
^2^ Vocabulary	--	0.3 **	0.25 **	0.34 **
zRecall		--	0.54 **	0.83 **
d-prime			--	0.91 **
Scomp				--

^1^ zRecall = standardized spelling recall scores; Scomp = a composite measure of spelling recall and recognition (d-prime) scores. ^2^ ** *p* < 0.001.

**Table 4:** Results of the overall LMMs and GLMMs for overall target word measures ^1^.

	Log FF			
**Predictor**	**b**	***SE***	***t***	***p***
(Intercept)	5.43	0.01	443.31	<1 × 10^−4^
**Freq ^2^**	**−0.02**	**0.01**	**−2.22**	**0.03**
Prime	0.00	0.00	−0.13	0.89
Freq:Prime	0.00	0.00	0.70	0.49
	**Log GD**			
	**b**	***SE***	***t***	***p***
(Intercept)	5.51	0.02	353.06	<1 × 10^−4^
**Freq**	**−0.04**	**0.01**	**−3.04**	**0.00**
Prime	0.00	0.01	−0.23	0.81
Freq:Prime	0.00	0.01	−0.23	0.82
	**RI Probability**			
	**b**	***SE***	***z***	***p***
(Intercept)	−2.09	0.12	−18.11	0.00
Freq	0.11	0.10	1.17	0.24
Prime	−0.02	0.05	−0.36	0.72
Freq:Prime	0.03	0.05	0.56	0.57

^1^ Freq = frequency condition; Prime = prime condition; Vocab = standardized vocabulary score; Spell = standardized spelling score. ^2^ Significant effects are in bold text.

**Table 5:** Main effects of frequency on target word reading times: mean (SE) ^1^.

Target Frequency	First Fixation (ms) *	Gaze Duration (ms) **	Total Time (ms) ^†^
High	237 (2.0)	257 (2.9)	343 (5.3)
Low	248 (2.0)	278 (2.9)	363 (5.2)

^1^ * *p* < 0.05; ** *p* < 0.001; ^†^ = trend toward significance.

**Table 6:** Results of the overall LMMs and GLMMs for post-target measures ^1^.

	Log GD			
**Predictor**	**b**	***SE***	***t***	***p***
(Intercept)	6.59	0.06	118.76	<1 × 10^−4^
Freq	−0.09	0.05	−1.77	0.08
Prime	0.00	0.01	0.42	0.68
Freq:Prime	−0.01	0.01	−1.31	0.19
	**Log TT**			
	**b**	***SE***	***t***	***p***
(Intercept)	−0.84	0.07	−12.50	<1 × 10^−4^
Freq	0.03	0.06	0.60	0.55
Prime	−0.04	0.03	−1.14	0.26
**Freq:Prime ^2^**	**0.07**	**0.03**	**2.08**	**0.04**
	**RO Probability**			
	**b**	***SE***	***z***	***p***
(Intercept)	0.09	0.11	0.75	0.45
Freq	0.05	0.06	0.74	0.46
Prime	−0.05	0.04	−1.31	0.19
Freq:Prime	0.04	0.04	1.01	0.31

^1^ Freq = frequency condition; Prime = prime condition; Vocab = standardized vocabulary score; Spell = standardized spelling score. ^2^ Significant effects are in bold text.

**Table 7:** Results of the LMMs for log-transformed first fixation duration on the target ^1^.

		Log FF			
Predictor	b	*SE*	*CI*	*t*	*p*
(Intercept)	5.44	0.01	[5.41, 5.46]	452.87	<1 × 10^−4^
**Freq ^2^**	**−0.02**	**0.01**	**[−0.03, −0.00]**	**−2.21**	**0.03**
Prime	0.00	0.01	[−0.01, 0.01]	−0.19	0.85
**Vocab**	**−0.02**	**0.01**	**[−0.04, −0.00]**	**−2.22**	**0.03**
**Spell**	**−0.03**	**0.01**	**[−0.05, −0.01]**	**−2.70**	**0.01**
Freq:Prime	0.00	0.01	[−0.01, 0.01]	0.27	0.79
Freq:Vocab	0.01	0.01	[−0.00, 0.02]	1.45	0.15
Prime:Vocab	0.00	0.01	[−0.01, 0.01]	−0.35	0.72
Freq:Spell	0.00	0.01	[−0.01, 0.01]	0.36	0.72
Prime:Spell	0.01	0.01	[−0.00, 0.02]	1.50	0.13
Vocab:Spell	−0.01	0.01	[−0.03, 0.01]	−1.27	0.20
**Freq:Prime:Vocab**	**0.01**	**0.01**	**[−0.00, 0.02]**	**1.89**	**0.06**
Freq:Prime:Spell	0.00	0.01	[−0.01, 0.01]	−0.32	0.75
Freq:Vocab:Spell	0.00	0.00	[−0.00, 0.01]	−0.29	0.77
Prime:Vocab:Spell	0.00	0.00	[−0.01, 0.01]	0.65	0.52
Freq:Prime:Vocab:Spell	0.01	0.00	[−0.00, 0.02]	1.36	0.17

^1^ Freq = frequency condition; Prime = prime condition; Vocab = standardized vocabulary score; Spell = standardized spelling score. ^2^ Significant effects are in bold text.

**Table 8:** Results of the LMMs for log gaze duration on the target ^1^.

		Log GD			
Predictor	b	*SE*	*CI*	*t*	*p*
(Intercept)	5.51	0.02	[5.48, 5.54]	353.06	<1 × 10^−4^
**Freq ^2^**	**−0.04**	**0.01**	**[−0.06, −0.01]**	**−2.99**	**0.00**
Prime	0.00	0.01	[−0.02, 0.01]	−0.50	0.62
**Vocab**	**−0.03**	**0.01**	**[−0.05, −0.01]**	**−2.54**	**0.01**
**Spell**	**−0.04**	**0.01**	**[−0.06, −0.02]**	**−3.19**	**0.00**
Freq:Prime	0.00	0.01	[−0.02, 0.01]	−0.58	0.56
Freq:Vocab	0.01	0.01	[−0.00, 0.02]	1.27	0.20
Prime:Vocab	0.00	0.01	[−0.01, 0.01]	−0.12	0.91
Freq:Spell	0.01	0.01	[−0.01, 0.02]	0.83	0.41
Prime:Spell	0.00	0.01	[−0.01, 0.01]	0.28	0.78
Vocab:Spell	−0.02	0.01	[−0.04, 0.00]	−1.61	0.11
**Freq:Prime:Vocab**	**0.01**	**0.01**	**[0.00, 0.03]**	**2.06**	**0.04**
Freq:Prime:Spell	−0.01	0.01	[−0.02, 0.01]	−0.88	0.38
Freq:Vocab:Spell	0.00	0.01	[−0.01, 0.02]	0.68	0.50
Prime:Vocab:Spell	0.01	0.01	[−0.01, 0.02]	1.11	0.27
Freq:Prime:Vocab:Spell	0.00	0.01	[−0.01, 0.02]	0.90	0.37

^1^ Freq = frequency condition; Prime = prime condition; Vocab = standardized vocabulary score; Spell = standardized spelling score. ^2^ Significant effects are in bold text.

**Table 9:** Results of the GLMMs for regressions into the target word ^1^.

		RI			
Predictor	b	*SE*	*CI*	*z*	*p*
(Intercept)	−2.11	0.12	[−2.34, −1.88]	−17.80	0.00
Freq	0.12	0.10	[−0.07, 0.32]	1.25	0.21
Prime	−0.01	0.05	[−0.11, 0.10]	−0.12	0.90
Vocab	0.10	0.08	[−0.05, 0.26]	1.28	0.20
Spell	−0.06	0.08	[−0.23, 0.10]	−0.77	0.44
Freq:Prime	0.04	0.05	[−0.06, 0.14]	0.75	0.45
Freq:Vocab	0.01	0.06	[−0.10, 0.12]	0.18	0.86
Prime:Vocab	−0.09	0.06	[−0.20, 0.02]	−1.56	0.12
Freq:Spell	0.02	0.06	[−0.10, 0.13]	0.27	0.79
Prime:Spell	0.01	0.06	[−0.10, 0.12]	0.22	0.82
Vocab:Spell	0.01	0.07	[−0.12, 0.15]	0.20	0.84
**Freq:Prime:Vocab ^2^**	**0.11**	**0.06**	**[0.01, 0.22]**	**2.05**	**0.04**
Freq:Prime:Spell	−0.04	0.06	[−0.15, 0.07]	−0.74	0.46
Freq:Vocab:Spell	−0.02	0.05	[−0.11, 0.08]	−0.36	0.72
Prime:Vocab:Spell	−0.02	0.05	[−0.11, 0.08]	−0.47	0.64
Freq:Prime:Vocab:Spell	−0.06	0.05	[−0.15, 0.04]	−1.21	0.23

^1^ Freq = frequency condition; Prime = prime condition; Vocab = standardized vocabulary score; Spell = standardized spelling score. ^2^ Significant effects are in bold text.

**Table 10:** Results of the LMMs for log total time on the target ^1^.

				Log TT	
Predictor	b	*SE*	*CI*	*t*	*p*
(Intercept)	5.71	0.02	[5.67, 5.76]	230.96	<1 × 10^−4^
Freq	−0.03	0.02	[−0.07, 0.00]	−1.72	0.09
Prime	0.00	0.01	[−0.02, 0.01]	−0.58	0.56
Vocab	−0.03	0.02	[−0.06, 0.01]	−1.50	0.13
Spell	−0.04	0.02	[−0.07, 0.00]	−1.84	0.07
Freq:Prime	0.00	0.01	[−0.01, 0.02]	0.45	0.65
Freq:Vocab	0.01	0.01	[−0.01, 0.02]	0.94	0.35
Prime:Vocab	−0.01	0.01	[−0.03, 0.00]	−1.38	0.17
Freq:Spell	0.01	0.01	[−0.01, 0.03]	1.23	0.22
Prime:Spell	0.00	0.01	[−0.02, 0.01]	−0.31	0.76
Vocab:Spell	−0.01	0.02	[−0.04, 0.02]	−0.51	0.61
**Freq:Prime:Vocab ^2^**	**0.02**	**0.01**	**[0.01, 0.04]**	**2.65**	**0.01**
Freq:Prime:Spell	0.00	0.01	[−0.01, 0.02]	0.24	0.81
Freq:Vocab:Spell	0.00	0.01	[−0.01, 0.02]	0.04	0.97
Prime:Vocab:Spell	0.01	0.01	[−0.01, 0.03]	1.08	0.28
Freq:Prime:Vocab:Spell	0.00	0.01	[−0.02, 0.01]	−0.32	0.75

^1^ Freq = frequency condition; Prime = prime condition; Vocab = standardized vocabulary score; Spell = standardized spelling score. ^2^ Significant effects are in bold text.

**Table 11:** Results of the LMMs for log gaze duration in the post-target region ^1^.

		Log FPT			
Predictor	b	*SE*	*CI*	*t*	*p*
(Intercept)	6.60	0.06	[6.48, 6.69]	118.85	<1 × 10^−4^
Freq	−0.09	0.05	[−0.17, 0.03]	−1.77	0.08
Prime	0.00	0.01	[−0.01, 0.02]	0.35	0.72
Vocab	0.04	0.03	[−0.02, 0.08]	1.64	0.10
**Spell ^2^**	**−0.08**	**0.03**	**[−0.13, −0.01]**	**−2.90**	**0.00**
Freq:Prime	−0.01	0.01	[−0.02, 0.01]	−1.00	0.32
Freq:Vocab	−0.01	0.01	[−0.03, 0.01]	−0.99	0.32
Prime:Vocab	−0.01	0.01	[−0.02, 0.01]	−1.37	0.17
Freq:Spell	−0.01	0.01	[−0.03, 0.01]	−0.80	0.43
Prime:Spell	0.01	0.01	[−0.01, 0.03]	1.26	0.21
Vocab:Spell	−0.03	0.02	[−0.08, 0.00]	−1.46	0.14
Freq:Prime:Vocab	−0.01	0.01	[−0.03, 0.01]	−1.22	0.22
**Freq:Prime:Spell**	**0.02**	**0.01**	**[0.00, 0.04]**	**2.14**	**0.03**
Freq:Vocab:Spell	0.00	0.01	[−0.01, 0.03]	−0.28	0.78
Prime:Vocab:Spell	0.00	0.01	[−0.03, 0.00]	0.40	0.69
Freq:Prime:Vocab:Spell	0.00	0.01	[−0.02, 0.01]	−0.43	0.67

^1^ Freq = frequency condition; Prime = prime condition; Vocab = standardized vocabulary score; Spell = standardized spelling score. ^2^ Significant effects are in bold text.

**Table 12:** Results of the GLMMs for regressions out of the post-target region ^1^.

		RO			
Predictor	b	*SE*	*CI*	*z*	*p*
(Intercept)	0.04	0.12	[−0.20, 0.27]	0.30	0.76
Freq	0.07	0.06	[−0.06, 0.19]	1.04	0.30
Prime	−0.05	0.04	[−0.12, 0.03]	−1.18	0.24
Vocab	−0.01	0.11	[−0.23, 0.20]	−0.12	0.90
Spell	0.14	0.11	[−0.08, 0.36]	1.27	0.20
Freq:Prime	0.05	0.04	[−0.03, 0.12]	1.16	0.25
Freq:Vocab	0.03	0.04	[−0.05, 0.10]	0.64	0.52
Prime:Vocab	−0.05	0.04	[−0.13, 0.02]	−1.35	0.18
Freq:Spell	0.08	0.04	[−0.01, 0.16]	1.81	0.07
Prime:Spell	−0.01	0.04	[−0.09, 0.07]	−0.20	0.84
Vocab:Spell	0.16	0.09	[−0.02, 0.34]	1.74	0.08
**Freq:Prime:Vocab ^2^**	**0.08**	**0.04**	**[0.01, 0.16]**	**2.10**	**0.04**
Freq:Prime:Spell	0.00	0.04	[−0.08, 0.08]	0.05	0.96
Freq:Vocab:Spell	−0.03	0.04	[−0.10, 0.04]	−0.97	0.33
Prime:Vocab:Spell	−0.01	0.04	[−0.10, 0.04]	−0.41	0.68
Freq:Prime:Vocab:Spell	−0.01	0.04	[−0.08, 0.05]	−0.42	0.67

^1^ Freq = frequency condition; Prime = prime condition; Vocab = standardized vocabulary score; Spell = standardized spelling score. ^2^ Significant effects are in bold text.

**Table 13:** Means and standard errors by vocabulary skill group for log gaze duration on the target.

		Low Frequency		High Frequency	
Vocabulary Skill	Total	Unrelated	Related	Unrelated	Related
Below Average	286 (5.0)	287 (8.7)	307 (11.1)	281 (11.5)	269 (8.6)
Average	270 (2.7)	282 (5.6)	281 (5.5)	259 (5.1)	252 (5.4)
Above Average	248 (3.9)	254 (7.3)	246 (7.2)	237 (8.7)	254 (8.2)

**Table 14:** Means and standard errors by vocabulary group for log total time on the target.

		Low Frequency		High Frequency	
Vocabulary Skill	Total	Unrelated	Related	Unrelated	Related
Below Average	381 (9.6)	359 (14.6)	430 (22.9)	370 (21.4)	367 (17.2)
Average	350 (4.7)	363 (10.1)	362 (8.9)	343 (9.2)	330 (8.9)
Above Average	336 (7.7)	359 (15.6)	313 (11.8)	322 (15.3)	350 (18.6)

## Data Availability

The original contributions presented in this study are included in the article. Further inquiries can be directed to the corresponding author.

## References

[1] Cain K., Oakhill J., Lemmon K. (2004). Individual differences in the inference of word meanings from context: The influence of reading comprehension, vocabulary knowledge, and memory capacity. J. Educ. Psychol..

[2] Castles A., Nation K., Snowling M.J., Hulme C. (2022). Learning to read words. The Science of Reading.

[3] Stanovich K.E. (1980). Toward an Interactive-Compensatory Model of Individual Differences in the Development of Reading Fluency. Read. Res. Q..

[4] Stanovich K.E., Nathan R.G., Vala-Rossi M. (1986). Developmental Changes in the Cognitive Correlates of Reading Ability and the Developmental Lag Hypothesis. Read. Res. Q..

[5] Zinar S. (2000). The relative contributions of word identification skill and comprehension-monitoring behavior to reading comprehension ability. Contemp. Educ. Psychol..

[6] Hart L., Perfetti C.A. (2008). Learning words in Zekkish: Implications for understanding lexical representation. Single Word Reading: Behavioral and Biological Perspectives.

[7] Perfetti C., Stafura J. (2014). Word knowledge in a theory of reading comprehension. Sci. Stud. Read..

[8] Stafura J.Z., Perfetti C.A. (2014). Word-to-text integration: Message level and lexical level influences in ERPs. Neuropsychologia.

[9] Ashby J., Rayner K., Clifton C. (2005). Eye movements of highly skilled and average readers: Differential effects of frequency and predictability. Q. J. Exp. Psychol..

[10] Ehrlich S.F., Rayner K. (1981). Contextual effects on word perception and eye movements during reading. J. Verbal Learn. Verbal Behav..

[11] Hess D.J., Foss D.J., Carroll P. (1995). Effects of global and local context on lexical processing during language comprehension. J. Exp. Psychol. Gen..

[12] Morris R.K. (1994). Lexical and message-level sentence context effects on fixation times in reading. J. Exp. Psychol. Learn. Mem. Cogn..

[13] Morris R.K., Folk J.R. (1998). Focus as a contextual priming mechanism in reading. Mem. Cogn..

[14] Simpson G.B., Peterson R.R., Casteel M.A., Burgess C. (1989). Lexical and sentence context effects in word recognition. J. Exp. Psychol. Learn. Mem. Cogn..

[15] Traxler M.J., Foss D.J., Seely R.E., Kaup B., Morris R.K. (2000). Priming in sentence processing: Intralexical spreading activation, schemas, and situation models. J. Psycholinguist. Res..

[16] Traxler M.J., Foss D.J. (2000). Effects of sentence constraint on priming in natural language comprehension. J. Exp. Psychol. Learn. Mem. Cogn..

[17] Foss D.J., Speer S.R., Hoffman R.R., Palermo D.S. (1991). Global and local context effects in sentence processing. Cognition and the Symbolic Processes: Applied and Ecological Perspectives.

[18] Staub A. (2015). The effect of lexical predictability on eye movements in reading: Critical review and theoretical interpretation. Lang. Linguist. Compass.

[19] Veldre A., Andrews S. (2018). Beyond cloze probability: Parafoveal processing of semantic and syntactic information during reading. J. Mem. Lang..

[20] Collins A.M., Loftus E.F. (1975). A spreading-activation theory of semantic processing. Psychol. Rev..

[21] Seidenberg M.S., Tanenhaus M.K., Leiman J.M., Bienkowski M. (1982). Automatic access of the meanings of ambiguous words in context: Some limitations of knowledge-based processing. Cogn. Psychol..

[22] Swinney D.A. (1979). Lexical access during sentence comprehension: (Re)consideration of context effects. J. Verbal Learn. Verbal Behav..

[23] Dell G.S., Reich P.A. (1981). Stages in Sentence Production: An Analysis of Speech Error Data. J. Verbal Learn. Verbal Behav..

[24] Meyer D.E., Schvaneveldt R.W. (1976). Meaning, Memory Structure, and Mental Processes. Science.

[25] Foss D.J. (1982). A discourse on semantic priming. Cogn. Psychol..

[26] Andrews S., Bond R. (2009). Lexical expertise and reading skill: Bottom-up and top-down processing of lexical ambiguity. Read. Writ..

[27] Andrews S. (2008). Lexical Expertise and Reading Skill. Psychol. Learn. Motiv..

[28] Perfetti C. (2007). Reading ability: Lexical quality to comprehension. Sci. Stud. Read..

[29] Perfetti C.A., Hart L. (2002). The lexical quality hypothesis. Precursors Funct. Lit..

[30] Myers J.L., O’Brien E.J. (1998). Accessing the discourse representation during reading. Discourse Process..

[31] Coltheart M., Rastle K., Perry C., Langdon R., Ziegler J. (2001). DRC: A dual route cascaded model of visual word recognition and reading aloud. Psychol. Rev..

[32] Rayner K. (1998). Eye movements in reading and information processing: 20 years of research. Psychol. Bull..

[33] Andrews S., Lo S., Xia V. (2017). Individual differences in automatic semantic priming. J. Exp. Psychol. Hum. Percept. Perform..

[34] Landi N., Perfetti C.A., Bolger D.J., Dunlap S., Foorman B.R. (2006). The role of discourse context in developing word form representations: A paradoxical relation between reading and learning. J. Exp. Child Psychol..

[35] Taylor J.N., Perfetti C.A. (2016). Eye movements reveal readers’ lexical quality and reading experience. Read. Writ..

[36] Andrews S., Reynolds G., Britt M.A., Goldman S.R., Rouet J.F. (2012). Why It Is Easier to Wreak Havoc than Unleash Havoc: The Role of Lexical Co-occurrence, Predictability, and Reading Proficiency in Sentence Reading. Reading–From Words to Multiple Texts.

[37] Bullinaria J.A., Levy J.P. (2007). Extracting semantic representations from word co-occurrence statistics: A computational study. Behav. Res. Methods.

[38] Lee C.E., Godwin H.J., Blythe H.I., Drieghe D. (2025). Individual differences in skilled reading and the word frequency effect. J. Exp. Psychol. Learn. Mem. Cogn..

[39] Brysbaert M., Stevens M. (2018). Power Analysis and Effect Size in Mixed Effects Models: A Tutorial. J. Cogn..

[40] Baayen R.H., Piepenbrock R., Gulikers L. (1996). The CELEX Lexical Database (CD-ROM). https://pure.mpg.de/pubman/faces/ViewItemOverviewPage.jsp?itemId=item_2339741.

[41] Landauer T.K., Dumais S.T. (1997). A solution to Plato’s problem: The latent semantic analysis theory of acquisition, induction, and representation of knowledge. Psychol. Rev..

[42] Eskenazi M.A., Folk J.R. (2015). Reading skill and word skipping: Implications for visual and linguistic accounts of word skipping. J. Exp. Psychol. Learn. Mem. Cogn..

[43] SR Research Ltd. (2013). EyeLink 1000 Plus.

[44] Brown J.I., Fishco V.V., Hanna G.S. (1993). Nelson-Denny Reading Test. Manual for Scoring and Interpretation, Forms G & H.

[45] Burt J.S., Tate H. (2002). Does a reading lexicon provide orthographic representations for spelling?. J. Mem. Lang..

[46] Eskenazi M.A., Askew R.L., Folk J.R. (2023). Precision in the measurement of lexical expertise: The selection of optimal items for a spelling assessment. Behav. Res. Methods.

[47] Hersch J., Andrews S. (2012). Lexical quality and reading skill: Bottom-up and top-down contributions to sentence processing. Sci. Stud. Read..

[48] Andrews S., Hersch J. (2010). Lexical precision in skilled readers: Individual differences in masked neighbor priming. J. Exp. Psychol. Gen..

[49] Slattery T.J., Yates M. (2018). Word skipping: Effects of word length, predictability, spelling and reading skill. Q. J. Exp. Psychol..

[50] Drieghe D., Rayner K., Pollatsek A. (2005). Eye Movements and Word Skipping During Reading Revisited. J. Exp. Psychol. Hum. Percept. Perform..

[51] R Core Team (2019). R: A language and Environment for Statistical Computing.

[52] Barr D.J., Levy R., Scheepers C., Tily H.J. (2013). Random effects structure for confirmatory hypothesis testing: Keep it maximal. J. Mem. Lang..

[53] Forster K.I. (1979). Levels of processing and the structure of the language processor. Sentence Process..

[54] Warren T., McConnell K., Rayner K. (2008). Effects of context on eye movements when reading about possible and impossible events. J. Exp. Psychol. Learn. Mem. Cogn..

[55] Eskenazi M.A., Folk J.R. (2017). Regressions during reading: The cost depends on the cause. Psychon. Bull. Rev..

[56] Rayner K., Warren T., Juhasz B.J., Liversedge S.P. (2004). The effect of plausibility on eye movements in reading. J. Exp. Psychol. Learn. Mem. Cogn..

[57] Warren T., McConnell K. (2007). Investigating effects of selectional restriction violations and plausibility violation severity on eye-movements in reading. Psychon. Bull. Rev..

[58] Wlotko E.W., Federmeier K.D. (2012). So that’s what you meant! Event-related potentials reveal multiple aspects of context use during construction of message-level meaning. NeuroImage.

[59] Duffy S.A., Morris R.K., Rayner K. (1988). Lexical ambiguity and fixation times in reading. J. Mem. Lang..

[60] van Berkum J.J.A., Brown C.M., Hagoort P. (1999). Early referential context effects in sentence processing: Evidence from event-related brain potentials. J. Mem. Lang..

[61] George M.S., Mannes S., Hoffinan J.E. (1994). Global semantic expectancy and language comprehension. J. Cogn. Neurosci..

[62] Rayner K., Sereno S.C., Lesch M.F., Pollatsek A. (1995). Phonological codes are automatically activated during reading: Evidence from an eye movement priming paradigm. Psychol. Sci..

